# Comparative study of audiometrics tests on metallurgical workers exposed to noise only as well as noise associated to the handling of chemical products

**DOI:** 10.1016/S1808-8694(15)30831-4

**Published:** 2015-10-18

**Authors:** Carla Tomaz Botelho, Anna Paulla Maia Lopes Paz, André Martins Gonçalves, Silvana Frota

**Affiliations:** 1 Speech therapist.; 2 Speech therapist.; 3 Work safety engineer.; 4 Doctorate in Human Communications Disorders. Speech therapist.; CEFAC - Centro de Especialização em Fonoaudiologia Clínica.

**Keywords:** hearing, occupational, hearing loss, chemical products, noise, solvents

## Abstract

Exposure to ototoxic chemical products in the presence or absence of noise can cause irreversible injury to the hearing of workers for a significantly short period of exposure period. **Aim:** to perform a comparative study, through audiometric tests, in workers exposed to noise only and noise associated with chemical products. **Materials and Methods:**155 steel workers (18 - 50 years) exposed to noise (group I), and exposed to noise and chemical products (group II) for a period that varies from 3 to 20 years. **Results:** significant difference in the rate of occupational hearing loss in the right ear between groups I (3.6%) and II (15.5%). A significantly higher rate of occupational hearing loss in group II (18.3%) and I (6%). With respect to the average time of exposure to aggressive agents, group I was exposed by a significantly higher time. Retrospective study. **Discussion:** The fact that the right ear was more affected in group II is controversial and should be better investigated in the future, since some studies show that the left ear would be more prone to noise-induced hearing loss. **Conclusion:** group II had proportionally a higher rate of hearing loss when compared with group I, even after having been exposed to aggressive agents for a lower average time.

## INTRODUCTION

We will use the term occupation hearing loss in this article, as a reference about the harm against hearing that the work environment can cause, regardless of noise.^1^

Among the factors that may results in occupational risk, noise certainly appears as the most frequent; many workers are exposed, especially steelworkers.^2^, ^3^, ^4^ There are, however, other factors that cause similar risks, such as ototoxic chemical products, which may cause occupational hearing loss in the presence or absence of noise.^1^, ^2^, ^5^, ^6^, ^7^, ^8^, ^9^, ^10^

There have been few epidemiological studies about the exposure time needed for causing hearing loss. Some researchers have stated that individuals in contact with ototoxic chemical products may start presenting hearing loss after two to three years of exposure to these substances, while individuals exposed to noise would take about four to five years to present similar symptoms. The frequency of compromised individuals increases after five to seven years; the progression of loss decreases until about 15 years, when it tends to stabilize as long as the exposure conditions persist and there are no additional causal factors.^2^, ^5^, ^6^

Prolonged exposure to noise and to chemical agents, however, do not make it certain that occupational hearing loss will occur; besides the environmental issues, there are also those pertaining to each individual, such as genetic factors, age, sex, and race, and endogenous factors linked to the causative agent, its form and occurrence, among others.^2^, ^5^

Exposure to chemical products currently remains undervalued; as a results noise is considered as the only cause of occupational hearing loss. It should be stated that occasionally noisy settings are not the main cause of hearing loss, giving place to places where workers are exposed to ototoxic chemical products.^6^, ^11^

Certain chemical products, such as solvents, heavy metals, asphyxiants, and more recently, the organophos-phorate pesticides, have been investigated for their potentially ototoxic effect, since these substances are found in a variety of production processes.^1^ Their molecular structures differ, and they can act on different parts of the auditory system in various ways.^5^

Organic solvents, especially toluene, xylene, styrene, n-hexane, trichloroethylene and ethanol are the most studied of this class; these are high priority ototoxic substances, but there are other that are less ototoxic such as ethyl acetate and carbon disulphide. All of these may be related to auditory disorders.^1^, ^2^, ^6^, ^12^, ^13^ There are some heavy metals in the list of other ototoxic substances, such as cobalt, mercury, benzene and arsenic.^12^

A study in a printing company in the state of Sao Paulo, based on a multiple logistic regression test, showed a high percentage of audiometric changes that occurred by exposure to ototoxic substances rather than by noise.^2^

A longitudinal study that surveyed industry workers exposed to chemical products during 20 years revealed that there was a high percentage of hearing loss, even though these workers had been exposed to low sound pressure levels.^12^

Experimental rats exposed to a mixture of styrene and xylene developed hearing loss; exposure to styrene caused permanent and progressive damage to their auditory system. This experiment suggested that these substances are more ototoxic than toluene, possibly due to the fact that they administered jointly, which may have facilitated absorption and increased the toxicity risk.^6^, ^14^

Another experiment in which rats were exposed to ethanol and styrene clearly demonstrated that the former substance caused no harm to the animal’s auditory system; styrene, on the other hand, permanently altered the threshold and caused further harm to the outer hair cells. Simultaneous exposure to two substances increase the effect on thresholds and the loss of hair cells, being more harmful.^15^, ^16^ Studies on rats are needed, since these animals have metabolic features in common with humans in terms of solvents.^8^

Based on research, we may state that simultaneous exposure to noise and ototoxic substances is synergic, where the effect of combined exposure is higher than the sum of the effect of each agent singly.^1^, ^2^, ^6^, ^7^, ^9^, ^12^, ^17^, ^18^ A study of three groups of workers in a glass fiber factory has confirmed this finding. The first of these three groups was exposed to styrene and noise, the second was exposed to noise only, and the third was not exposed to either agent. Commenting this study based on the results of the present study, we may state that pure tone thresholds at 2 000 to 6 000 Hz worsened in first group, compared to the other two groups. We may also state that styrene is toxic to the auditory system even when present below the recommended tolerance levels.^19^

Workers were divided into four groups in a study done at a specific industry. The first group was exposed to a mixture of organic solvents, the second group was exposed to the same mixture and also to noise above 80 dBHL, the third group was exposed to neither (and was used as the control group), and the fourth group was exposed to noise above 80 dBHL only. Compared to the control group, the highest prevalence of hearing loss was found in the first and second groups, particularly at 3 000 to 8 000 Hz. Hearing loss in the fourth group was concentrated at 4 000 Hz. There was a positive correlation among the dose accumulation of organic solvents, noise, and the degree of hearing loss.^15^

We thus need to reflect on the tolerance levels to various agents in the working environment; these may be present within acceptable parameters, as established by Brazilian law for each substance singly, but may become unacceptable when combined, which reinforces their effect on the auditory system.^6^, ^9^, ^20^, ^21^

Hearing loss due to ototoxic chemical substances has similar audiometric features compared to hearing loss due to noise. It is always sensorineural and irreversible, and may be unilateral or bilateral.^1^, ^2^, ^5^, ^20^, ^22^ High frequencies are affected first - particularly at 4 000 Hz - since injury concentrates on the basal turn of the cochlea; it takes time for hearing loss to extend beyond the 3 000 Hz to 6 000 Hz range.^2^

Noise and chemical substances may injure the cochlea;^23^, ^24^ when referring to the injury site of chemical substances, however, we may state than ototoxic effects are not restricted only to the cochlea. Many organic solvents are neurotoxic, and may affect hearing and balance, causing harm initially to the brainstem or the central auditory/vestibular pathways.^5^, ^12^ Solvent-caused vestibular dysfunction has been neglected due to assessment difficulties in this field.^17^

In rats, aromatic solvents - such as styrene - appear to decrease the auditory sensitivity, particularly at middle frequencies. These substances may cause permanent hearing loss by affecting first the outer hair cells, and eventually the spiral ganglia.^17^

A few researchers have shown that exposure to toluene, styrene and carbon monoxide may alter the function and morphology of outer hair cells in experimental animals.^2^, ^23^

Other clinical and epidemiological studies support us in stating that there is an association between various solvents and changes in central auditory pathways; based on audiometric findings, we may also perceive that these substances cause mild to moderate hearing loss.^25^

A survey was made of workers of a given industry that were exposed to a mixture of aromatic and aliphatic solvents. The results revealed a low speech recognition rate in voice audiometry compared to pure tone audiometry, and that cortical responses were altered at the frequencies that were tested. The conclusion was that the auditory system might be vulnerable at a cortical level, which was confirmed by speech discrimination tests and cortical responses, two of the most sensitive tests available for detecting cortical central auditory injuries.^12^

Annex I of the Regulation Norm 15 defines the tolerance levels for continuous and intermittent noise. However, the specific Brazilian labor laws do not state that periodic audiometric testing should be done in workers exposed to ototoxic chemicals; periodic audiometric testing is required only of workers exposed to noise levels above 85 dBHL 8 hours per day.^1^, ^6^, ^20^

In 1998, the North-American army started taking exposure to chemical substances into account in the prevention of hearing loss; this extended to workers exposed to certain substances such as styrene, which were included in hearing conservation programs regardless of their exposure level to noise.^26^

Research institutions, such as the NIOSH and the ACGIH, have also recommended audiometric testing of these workers as of 1998.^27^, ^28^

The Social Security Law 3048 of 6 May 1999 opened the possibility of recognizing certain chemical substances as occupational etiological agents or risk factors for ototoxic hypoacusis; thus, this type of exposure should be taken into account when searching for a cause-effect relation between hearing loss and the work environment.^29^

The European Parliament has recently started requiring that employers pay attention to the effects of the interaction among noise, chemical substances and health.^30^

Comparing the general features - such as audiometric tracings and the affected site - of hearing loss induced by noise and ototoxic chemical substances raises awareness of the difficulties in making a differential diagnosis and identifying the cause. The diagnosis is based on confirming the exposure and finding signs and symptoms, even though the effects are poorly established.^5^, ^10^

The purpose of this study was to compare auditory thresholds, by using audiometric tests, of two groups of workers, one exposed only to noise and another exposed to noise and chemical substances.

## METHOD

A case-control study was undertaken of 155 workers in a steel company in the state of Rio de Janeiro. All subjects were male; 81 were allocated to group I (exposure to noise), and 71 to group II (exposed to noise and chemical substances). The mean age was 31 (+/- 7) years, ranging from 18 to 50 years; the mean general exposure time was 7 (+/- 4) years, ranging from 3 to 20 years.

The chemical substances that were assessed were: acetone, styrene, resins, and cobalt, among others of less relevance.

The study was done in different sectors of the factory, in which noise ranges from 80.5 dBHL to 99.5 dBHL, and where exposure time is 8 hours per day. Workers used individual protection equipment as required by the Regulation Norm 6,20 Law 3214/78; of relevance to our study is the use of adequate breathing masks and ear protectors, both certified by the Ministry of Labor and Employment. The company is committed to the physical integrity of its employees, and its technical unit effectively inspects the use of safety equipment, with penalties - as defined by law - for non-compliance.

Trained professionals employed by the company measured the noise levels with company-owned environmental monitoring equipment. Measurements were done at different sites of the working area on the A-scale, slow-response circuit, as required by the Regulation Norm 15, annex number 1, Law 3214/78.20 A Quest model Q-400 dosimeter, duly calibrated according to the norm IEC 60651, procedure PC-06 - REV 00, October 2005, was used.

The results of organic vapor evaluations in the samples were lower than the limits required by the Regulation Norm 15, annex 11, Law 3214/78.20 PVC-filter cassettes were used, and a certified specialized laboratory issued the technical reports.

A retrospective study of sequential audiometric tests in 2005, carried out by a trained professional: a speech therapist according to the Federal Medical Board and the Speech Therapy Federal Board and according to the requirements contained in the Law 19 of the Ministry of Labor and Employment (1998).^31^

Tests were done in an audiometry test suite wherein the sound pressure was within the maximum limits required by the ISO 8253.1 Norm; the audiometer was a MAICO MA 41 device, which is calibrated electroacoustically once a year.

All workers had a 14-hour auditory rest period before testing; all underwent meatoscopy of both ears before testing. Those with abnormal external acoustic meatuses were excluded from the study and referred for healthcare.

Audiometric tests were done at 500, 1 000, 2 000, 3 000, 4 000, 6 000 and 8 000 Hz (air conduction), and 500, 1 000, 2 000, 3 000 and 4 000 Hz (bone conduction) if air conduction results were altered. Voice audiometries were also done in all subjects (speech reception threshold and speech recognition score).

Occupational hearing loss was considered as present when auditory thresholds were over 25 dBHL at 3 and/or 4 and/or 6 KHz, above other tested frequencies, whether altered or not, in both air and bone conduction, in one or both sides.^31^

The Research Ethics Committee approved this study (number 127/06).

**Chart 1:**
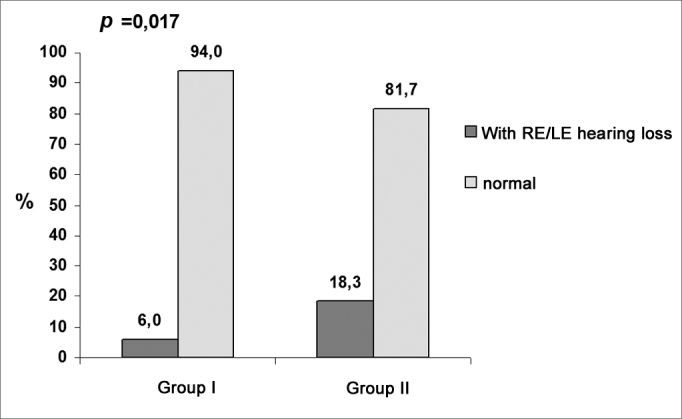
Prevalence of hearing loss related to professionals (right or left ears).

**Chart 2:**
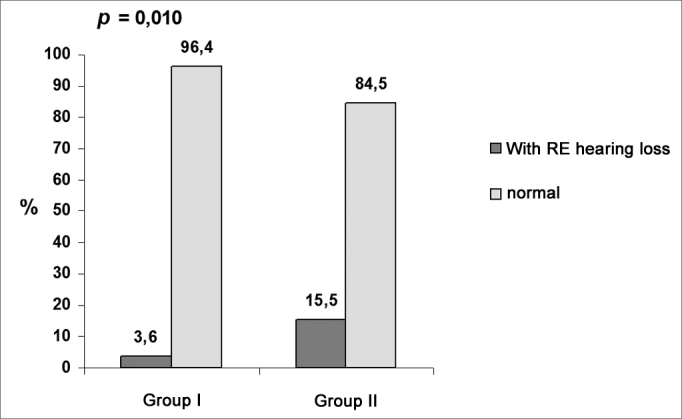
Prevalence of hearing loss related to the right ear.

**Chart 3:**
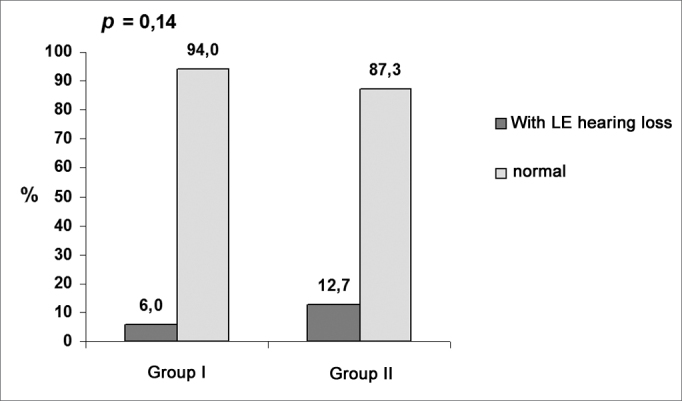
Prevalence of hearing loss related to the left ear.

The statistical analysis consisted of the chi-square (χ2) test for comparing occupational hearing loss between both groups, Student’s t test form independent samples (age) for comparing the age (in years) and Mann-Whitney’s test for comparing the duration of exposure (in years). Qualitative data were summarized by frequency (n) and percentages (%). Charts 1, 2 and 3 illustrate the prevalence of hearing loss among the factory sectors. Numeric data were expressed as measures of central tendency (mean and median) and dispersion (standard deviation). A 5% significance level was adopted; a p value equal to or below 0.05 was statistically significant.

## RESULTS

Data on Table 1 shows that there was a significant difference (p = 0.010) in the proportion of occupational hearing loss in the right ear between groups I (3.6%) and II (15.5%). There was no significant difference (p = 0.14) in occupational hearing loss in the left ear between groups I (6%) and II (12.7%). Additionally, there was a significant difference in the proportion of occupational hearing loss among professionals (right and left ears) between groups I (6%) and II (18,3%).

**Table 1:** Percentage of occupational hearing loss in groups I and II.

		GROUP I		GROUP II		
Variable	class	n	%	n	%	p value
Occupational hearing loss in the right ear	yes no	3 81	3,6 96,4	11 60	15,5 84,5	0,010
Occupational hearing loss in the left ear	yes no	5 79	6,0 94,1	9 62	12,7 87,3	0,14
Occupational hearing loss in the right and left ears	yes no	5 79	6,0 94,1	13 58	18,3 81,7	0,017

Chi-square test; p ≤ 0.05 - statistically significant

Data on Table 2 shows that there was no significant difference (p = 0.26) in the mean age between both sectors. There was a significant difference (p = 0.0003) in the mean duration of exposure; group I had a significantly higher exposure time than group II. Based on these findings, we may state that hearing loss has a different statistical behavior in each sector; thus, there was no sense in considering both ears jointly, but rather to measure hearing loss related to each professional.

**Table 2:** Statistical analysis of the age and duration of exposure in groups I and II.

Variable	Sector	n	Mean	SD	Median	Minimum	Maximum	p value
Age	Group I	84	30,5	6,8	29	18	45	
(in years)	Group II	71	31,8	7,5	30	19	50	0,26
Duration of exposure	Group I	84	7,6	3,5	6	3	20	
(in years)	Group II	71	6,1	3,3	5	3	20	0,0003

SD: Standard deviation

Student’s t test; p ≤ 0.05 - statistically significant

The Mann -Whitney test; p ≤ 0.05 - statistically significant

## DISCUSSION

Table 1 reveals a higher percentage of occupational hearing loss in group II; thus, we may suggest that the combination of noise and chemical substances causes a higher incidence of hearing loss.^15^, ^19^

The fact that the right ear was more affected in group II deserves further discussion, since opinions vary in different studies. Some researchers present data showing that there is a prevalence of occupational hearing loss in one ear when ears are compared. This may occur, for instance, when a machine is located next to a specific side of the worker, or when only one side of the work site in which workers undertake their activities is open; these conditions were not present in the steel mill in question.^32^ Results are controversial even in these cases,^33^ since, according to various authors, many factors may affect these results; one of them is individual susceptibility.^34^

A few studies have suggested that the left ear is more susceptible to noise injury, although evidence for this statement is scarce. Other equally important studies have shown that male adult hearing is about 4 dB (HL) lower in the left ear compared to the right ear.^32^ These findings describe the opposite of what we found in our study. Thus, additional research is needed.

Table 2 shows a lower mean duration of exposure in group II compared with group I until the onset of occupational hearing loss, reinforcing the first idea that exposure to these agents in combination increases the effect on hearing.

Audiometric testing is essential for both the prevention and monitoring of occupational hearing loss. Also needed is a hearing conservation program for workers exposed to chemical substances, with or without exposure to noise levels over 85 dB.

## CONCLUSION

Group II had a higher prevalence of occupational hearing loss compared to group I, although the latter was exposed to the aggressive agent for a longer period.
